# Hazardous Chemicals Associated with Plastics and Plastic Waste: Environmental Occurrence and Toxicity of TCDD, DEHP, BPA, and Acrylonitrile

**DOI:** 10.3390/ma19204368

**Published:** 2026-10-17

**Authors:** Maciej Kozłowski, Małgorzata Zawadzka, Monika Kalinowska, Marzena Matejczyk

**Affiliations:** Department of Chemistry, Biology and Biotechnology, Institute of Civil Engineering and Energetics, Faculty of Civil Engineering and Environmental Science, Bialystok University of Technology, Wiejska 45E Street, 15-351 Bialystok, Poland; malgorzata.zawadzka@pb.edu.pl (M.Z.); m.kalinowska@pb.edu.pl (M.K.); m.matejczyk@pb.edu.pl (M.M.)

**Keywords:** acrylonitrile, bisphenol A, DEHP, persistent organic pollutants, POPs, TCDD

## Abstract

Hazardous compounds including 2,3,7,8-tetrachlorodibenzo-p-dioxin (TCDD), bis(2-ethylhexyl) phthalate (DEHP), bisphenol A (BPA), and acrylonitrile (ACN) are ubiquitous in environmental matrices such as soil and water. This review synthesizes their environmental occurrence and multi-organ toxicity. Its objective was to integrate occurrence and toxicity data for TCDD, DEHP, BPA, and ACN into a single account and to identify early-hazard biomarkers and regulatory gaps. A structured documentary search covering mainly 2000 to 2024 was performed in Google Scholar, ScienceDirect, Scopus, Web of Science, and PubMed, prioritizing in vivo and in vitro experimental studies and complemented by governmental and institutional reports. The analysis shows that all four compounds act through convergent mechanisms centered on oxidative stress and mitochondrial dysfunction, via compound-specific signalling pathways, and that adverse effects arise across cytotoxic, neurotoxic, cardiotoxic, reproductive, and immune endpoints at environmentally relevant concentrations, with some reproductive effects extending across generations. HSP47 and HSP70 emerge as early biomarkers of cellular stress. Although regulation has measurably reduced some exposures, chronic low-dose risk remains underestimated, supporting harmonized limits, premarket screening of substitutes, and source control.

## 1. Introduction

Synthetic chemicals such as plasticizers, breakdown products of plastic materials, and additives used in the production of plastic pose significant threats to human health and the natural environment. These toxicants enter the environment through various pathways, including leaching from landfills into soil and groundwater, contaminating surface waters via runoff from urban areas and industrial sites, and being released into the atmosphere during the production, use, and disposal of plastic products [1,2].

More than 13,000 chemicals have been identified as associated with plastics and plastic production across a wide range of applications [3]. Global production of plastics reached 430.9 million tonnes (Mt) in 2024, against a cumulative total of approximately 8300 Mt of plastics produced since mass production began. Of the plastic waste generated by 2015, around 9% had been recycled, 12% incinerated, and 79% accumulated in landfills or the natural environment [4,5]. Current projections indicate that plastics use could almost triple by 2060, rising from 460 Mt in 2019 to 1231 Mt, and that plastic waste will rise almost in parallel, while leakage of plastics into the environment is projected to double to 44 Mt per year [6].

2,3,7,8-tetrachlorodibenzo-p-dioxin (TCDD) is a highly toxic dioxin that is not intentionally used as a raw material or additive in plastic manufacturing. Instead, it may be formed unintentionally as a by-product of certain industrial and thermal processes, including waste incineration and processes involving chlorinated materials [7]. Di(2-ethylhexyl) phthalate (DEHP) is commonly used as a plasticizer in polyvinyl chloride (PVC) products, including toys, medical devices, building materials, and capacitors. Its leaching from products into the environment results in exposure through ingestion, inhalation, and dermal contact. DEHP has been linked to adverse effects on the reproductive system due to its being an endocrine disruptor [8]. Bisphenol A (BPA) is found in polycarbonate plastics, epoxy resins, and thermal paper receipts because of its application as a comonomer in the production of polycarbonates. BPA mimics hormones, potentially causing reproductive, metabolic, and developmental issues, even at low levels [9]. Acrylonitrile (ACN), which is employed in plastics, synthetic fibers, and rubber production, contaminates the environment during manufacturing and disposal. Most human exposure occurs in workplaces in factories that produce it. Exposure to ACN is linked to increased neurological and respiratory risks of cancer and reproductive disruption [10]. The compounds selected for this review represent different categories of hazardous substances associated with plastics and plastic waste rather than a chemically or functionally homogeneous group. DEHP is primarily used as a plasticizer, BPA is an important component or precursor in the production of polycarbonate and epoxy-based materials, and ACN is an industrial monomer used in the production of several polymeric materials. In contrast, TCDD is not intentionally used as a raw material or additive in plastic manufacturing. TCDD was included because it represents a highly toxic persistent contaminant that may be unintentionally formed during certain industrial and thermal processes, including processes associated with the treatment and combustion of chlorinated materials and plastic waste [11]. Numerous international government agencies monitor these pollutants in air, water, soil, and biological samples, among other media [12,13]. The goal of this ongoing surveillance is to determine how common they are, evaluate the risks, and put protective measures in place. For example, part of the Canadian Health Measures Survey (CHMS) was used to measure the environmental contents of some of those compounds. These levels gradually decrease; however, dioxins and furans are still present in the environment [13].

The need to comprehend and address the effects of such compounds on human health and the environment is the reason for the continuous research and monitoring of these substances [13,14]. Because of their longevity and capacity to bioaccumulate and biomagnify in food chains, even brief exposures can have a substantial cumulative impact over time. With respect to the aim of protecting the public’s health, comprehending the mechanisms of toxicity and the exposure pathway is essential for designing efficient risk assessment and management techniques. Furthermore, exposure to plastic is widespread and impacts a variety of people globally due to its broad use in numerous industrial, agricultural, and consumer applications. Children, expectant mothers, and indigenous people are among the vulnerable groups that may be especially vulnerable because of higher exposure levels or heightened vulnerability to the harmful effects of these substances. The aim of this review is to integrate occurrence and multi-organ toxicity data for all four compounds in a single account, relate them to recent global projections of plastic production and waste, and identify monitoring biomarkers.

## 2. Methodology

The scientific literature was retrieved from international databases, including Google Scholar, ScienceDirect, Scopus, Web of Science, and PubMed. Full-length original research articles, review papers, in vivo and in vitro studies, and additionally selected governmental and institutional reports were included. Most of the toxicological literature analyzed in this review covers publications from 2000 to 2024. However, earlier studies were also included when justified, particularly those addressing contamination events such as Agent Orange-related TCDD exposure or long-term environmental monitoring in soil, water, and sediment matrices conducted in the 1980s and 1990s. Following search queries were constructed using compound-specific and toxicity-related keywords: search terms included (persistent organic pollutants or TCDD or 2,3,7,8-tetrachlorodibenzo-p-dioxin or dioxins or bisphenol A or BPA or di(2-ethylhexyl) phthalate or DEHP or acrylonitrile or ACN) AND (cytotoxicity or neurotoxicity or cardiotoxicity or reproductive toxicity or immunotoxicity or environmental occurrence). We prioritized experimental studies employing both animal models and cellular systems, as they constitute the core evidence base for mechanistic toxicology. In addition, environmental data derived from governmental and regulatory reports concerning contamination in soil, water, and sediments were included to provide contextual background on exposure sources and distribution. Information obtained from the selected publications was organized according to the four compounds considered in this review: TCDD, DEHP, BPA, and ACN. The collected data were first divided into information concerning their environmental sources and occurrence and information related to their toxicological effects. The toxicological evidence was subsequently grouped according to the main types of toxicity addressed in the literature, including cytotoxicity, neurotoxicity, cardiotoxicity, reproductive toxicity, and immunotoxicity. In addition, studies describing molecular mechanisms associated with heat shock proteins and carcinogenesis were considered separately.

## 3. Results and Discussion

### 3.1. Sources and Occurrences in the Environment

#### 3.1.1. 2,3,7,8-Tetrachlorodibenzo-p-dioxin

TCDD is a persistent environmental toxicant formed as a byproduct of industrial processes, including incineration, metal production, and paper and pulp bleaching, as mentioned in the National Toxicology Program (NTP) Report on Carcinogens (2021) [15]. Another significant source is the pulp and paper industry, where wood pulp is bleached with chlorine, leading to the formation of byproducts such as TCDD [16]. During the Vietnam War (1965–1972), 80 million liters of Agent Orange, containing the toxic dioxin TCDD, were sprayed to defoliate jungles, riverbanks, and crops in South Vietnam [14]. Previously, TCDD was also used as a conventional herbicide, for example, in New Zealand, where research has demonstrated a significant impact on the health of production workers [17], or in China, mainly as a fungicide and wood preservative [18].

TCDD is highly resistant to biodegradation, with half-lives ranging from less than a year to twelve years in soil interiors [19]. Currently, TCDD is more of a contaminant than a deliberate chemical. The focus of effort is to reduce its release into the environment and minimize its effects by means of strict regulations and pollution control strategies. Even though TCDD is not absorbed by plants, it can stick to surfaces such as soil particles and foliage. It accumulates in aquatic species and magnifies through the food chain, posing environmental and health risks [14]. When released into water, TCDD strongly adsorbs to sediments and suspended matter. Despite their low solubility in water (0.2 µg/L), other organic compounds in water can serve as carriers [19]. Its persistence is attributed to resistance, limited access to biodegradation and photodegradation, and slow volatilization caused by low vapor pressure [18].

**Figure 1:**
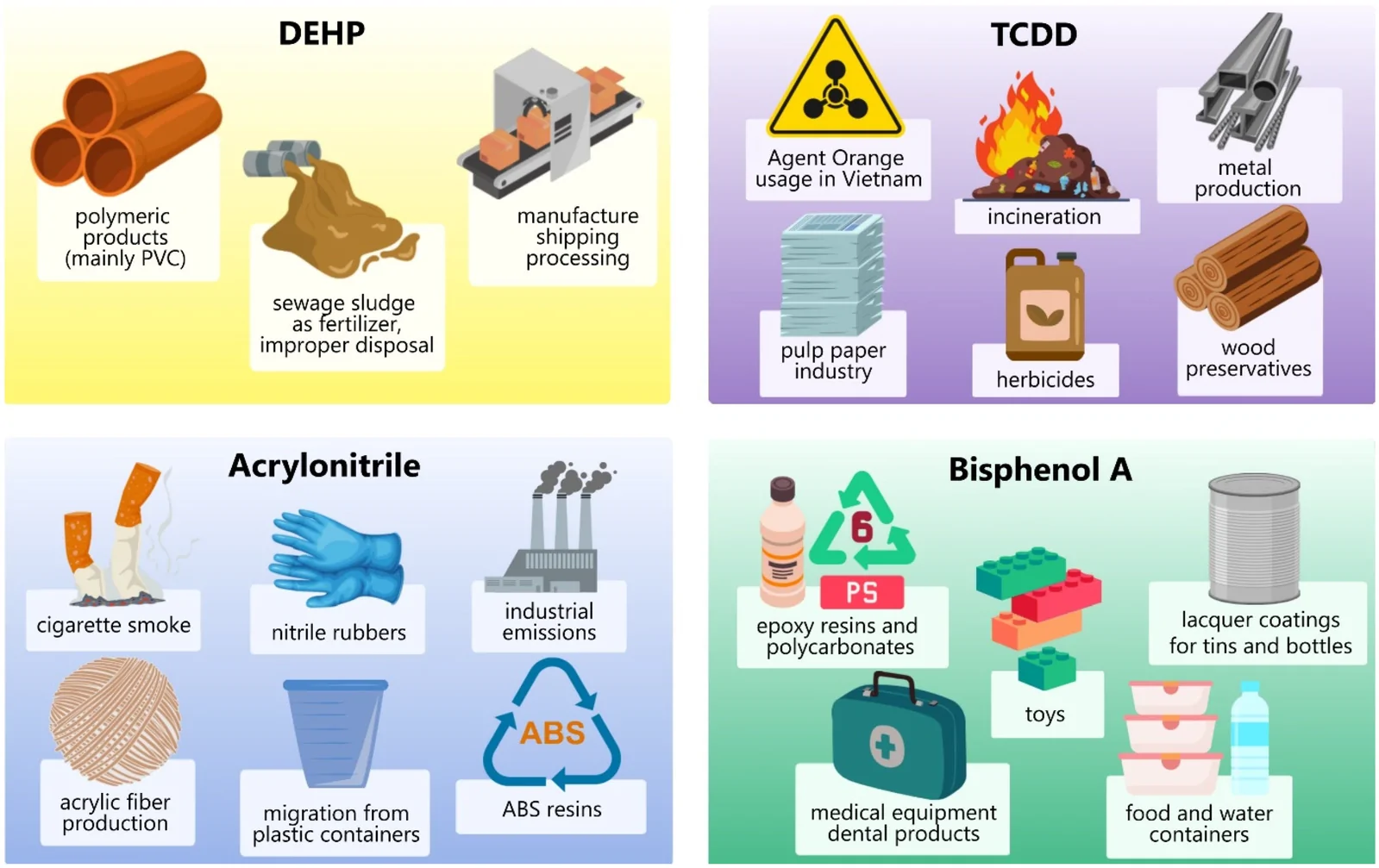
Main sources of selected toxins.

Soil and sediment samples were taken from the Bien Hoa Airbase in 1999, and research conducted revealed dioxin concentrations of up to 1,164,699 pg/g (ppt) [20]. In 2004, nearby areas presented contamination levels that were 20.4 times greater for sediment and 46 times greater for soil than standard values [21]. In 2017, topsoil from the A-So airbase still presented elevated TCDD levels more than fifty years after Agent Orange usage [22]. Reports on 2016–2020 dioxin levels in Hong Kong have shown that dioxin levels generally tend to decrease and that dioxin levels during the winter months are generally higher than those during the summer months [23]. Based on animal toxicity research, Kimbrough et al. suggested that a concentration of 1 ng/g TCDD in soil “is a reasonable level at which to begin considerations of action to limit human exposure to contaminated soil” [24]. Nestrick et al. analyzed soils from industrialized areas of a group of cities from the Midwestern and Mid-Atlantic states [25]. The concentrations of TCDD measured in this study ranged from ND (not detected) to 9.4 ppt. The soil samples were also gathered in England, Wales, and lowland Scotland, and the research confirmed the presence of TCDD at levels ranging from <0.5 to 2.1 ppt [26]. Various industrialized nations face dioxin-contaminated drinking water. In Ufa, Russia’s Bashkortostan Republic, industrial activities have polluted the water supply for decades. The city, with its numerous factories, has experienced incidents of the release of TCDD and other polychlorinated dibenzodioxins (PCDDs) into the Ufa River. Regular PCDD levels ranging from 0.13 to 0.20 ng/L (ppt) have been observed in rivers [27]. Emergency situations, where dioxin concentrations exceed the permissible level of 0.02 ng/L by ten to one hundred times, are common. Occupational exposure to TCDD, such as through Agent Orange usage in Vietnam, mainly occurs via inhalation and skin contact. Numerous studies have investigated Vietnam veterans exposed to Agent Orange. The background group had an average serum TCDD concentration of 5.8 ppt. In comparison, the exposed groups had significantly higher mean serum concentrations: 10–25.6 ppt in the low-exposure group and 18–617.8 ppt in the high-exposure group [28]. The main sources and environmental occurrence of TCDD are summarized in Table 1 and Figure 1.

#### 3.1.2. Bis(2-ethylhexyl) Phthalate

DEHP is extensively employed as a plasticizer in polymeric products. It is present in variable concentrations in PVC, ranging from 10 to 40%, contingent on the specific type of product [29]. It is produced in large quantities, with production and/or use in the United States reported at 45,000–230,000 tons in 2006 [30], in the European Union estimated at 500,000 tons per year [31], and global production reaching approximately 2 million metric tons in 2004 [32]. DEHP comes primarily from indoor and outdoor products such as PVC and is present in a variety of environmental matrices, including soil, sediments, and water bodies [30]. Moreover, the manufacture, shipping, processing, and improper disposal of DEHP all contribute to its environmental discharge [33]. With possible spread to air or water, its primary impact is on soil.

The DEHP concentrations in surface water vary from undetectable to 97.8 mg/L, whereas those in groundwater range from undetectable to 5.661 mg/L [30]. The distribution of DEHP in groundwater is influenced by surface water concentrations, and its presence has been identified in drinking water globally (Germany and Poland 0.05–0.06 mg/L, Greece 0.93 mg/L, USA 0.55 mg/L; China 3.47 mg/L) [34,35,36,37]. DEHP concentrations in aquatic environments exhibit country-specific variations, which are likely linked to the intensity of DEHP usage and regulatory practices [30]. Soil concentrations range from 1 to 264 mg/kg dry weight, with elevated levels observed near industrial sites [30]. The application of sewage sludge as fertilizer represents a potential source of DEHP accumulation in agricultural soil. Data provided by Health Canada indicate a decrease in DEHP concentrations in Canadians’ urine since these measures were implemented, with levels decreasing from 42 µg/L (2007–2009) to 9.5 µg/L (2018–2019) [12]. The National Health and Nutrition Examination Survey (NHANES) conducted biomonitoring to estimate exposure in the U.S. population aged 6–59. DEHP metabolites were measured in the urine samples, with corrections for urine dilution. Data from the NHANES programs from 1999 to 2012 revealed a decrease in the levels of DEHP metabolites, particularly mono(2-ethyl-5-hydroxyhexyl) phthalate (MEHHP), mono(2-ethyl-5-oxohexyl) phthalate (MEOHP), and mono(2-ethyl-5-carboxypentyl) phthalate (MECPP), although mono-2-ethylhexyl phthalate (MEHP) remained a minor component. The decline is consistent across age, sex, and ethnicity [38]. The European Human Biomonitoring Initiative (2014–2021) reported that the current concentrations of DEHP in children are all lower than the values reported by the DEMOCOPHES project for children aged 5–11 years (2011–2012) [39]. Qu et al. analyzed studies that spanned from 1982 to 2017 and reported a global decrease in DEHP exposure levels over time, from 4.40 μg/kg body weight/day before 2000 to 2.23 μg/kg bw/day from 2015 to 2017. Europe and Asia experienced fluctuating trends, whereas North America maintained relatively lower levels. These factors contributed to the decline, including usage restrictions, changes in production/consumption levels, and decreases in environmental concentrations. The EU directive banning DEHP in toys in 1999 led to a sharp decrease in children’s exposure. However, subsequent production and consumption peaks in the EU correlated with exposure increases. A comparison of DEHP restrictions with children’s exposure trends in the USA and Taiwan, China, revealed a delayed response to regulation due to slow DEHP release from products with long service lives [40]. The main sources and environmental occurrence of DEHP are summarized in Table 1 and Figure 1.

#### 3.1.3. Bisphenol A

With the production of epoxy resins and polycarbonates accounting for approximately 95% of its output, BPA is a major player in the global industrial sector. The annual production of BPA worldwide exceeds 3.8 million tons, and over the same period, 100 tons are released into the environment [41]. Synthetic polymers derived from BPA are valued for their excellent mechanical properties, low moisture adsorption, and thermal stability. These polymers are extensively employed in the production of diverse items, such as water pipes, food containers, bottles, toys, nipples, medical equipment, dental products, electronic devices, and CD/DVD discs [42]. Additionally, BPA plays a vital role in the manufacture of polyacrylates, polyesters, and lacquer coatings for tins, water bottles, and food packaging, which, upon degradation, may contribute significantly to environmental and food contamination [9]. BPA exposure primarily occurs through food consumption. This occurs when people eat or drink food that has been kept in recycled bottles, epoxy-lined cans, or polycarbonate containers where BPA has leached into the contents. The ester bonds that connect BPA molecules to epoxy resins and polycarbonates hydrolyze more rapidly in environments with high temperatures and high acidity. Low-pH polycarbonate solutions are more likely to contain BPA leakage, but epoxy resins are more susceptible to BPA leakage at higher temperatures [43].

BPA can be found in various environmental matrices, including air, sediment/soil, and water/wastewater (Table 1). Studies conducted in urban areas of India, China, New Zealand, the US, and Japan have shown high levels of BPA in PM10 aerosols, ranging from 0.2 to 17.4 ng/m^3^ [44]. BPA is prevalent in soil across various regions globally, with significant concentrations observed in Asian countries. China’s urban areas, which span 21 provinces, have reported BPA levels of approximately 166.0 ng/g. Urban sites in Chennai, India, present a wide range of BPA concentrations, from 200 to 17,400 ng/g, whereas Beijing’s urban sites present concentrations ranging from 380 to 1260 ng/g. Research conducted on sediment collected from the Gulf of Gdańsk revealed that the highest recorded concentration of BPA was 21.25 ng/g dry weight [45]. Studies have reported high BPA levels in surface water samples near industrial and sewage treatment plant discharge points, such as those in Shizuoka paper recycling plants in Japan, where concentrations range from 8000 to 370,000 ng/L. Furthermore, BPA was detected in European rivers, such as the Elbe in Germany, the Tiber, the River Schelde, and the Lambro, at concentrations ranging from 3 to 776 ng/L [46]. The main sources and environmental occurrence of BPA are summarized in Table 1 and Figure 1.

#### 3.1.4. Acrylonitrile

Acrylic fibers, acrylonitrile butadiene styrene (ABS) resins, adiponitrile, acrylamide, nitrile rubber, and carbon fibers are the primary sources of ACN. Acrylic fibers, which are primarily employed in apparel and home furnishings, represent the largest commercial use of acrylonitrile [47]. While the growth of the acrylic fiber market has slowed compared with that of other applications, its manufacturing capacity has shifted to Asia, particularly China [48].

ABS resins are used in electronics, appliances, construction, and automotive industries because of their high durability and coloring characteristics. Furthermore, ABS has recently been used in 3D printing [47]. Owing to their flexibility and resilience to chemicals, nitrile rubbers are mostly used in the production of electrical wire jackets, hoses, belts, gaskets, and seals [49]. Moreover, Wang et al. proposed a migration model of ACN from ABS plastic toys, which revealed a possible risk of exposure for children [50]. ACN is present in cigarette smoke, which is deemed the primary source of exposure to this xenobiotic outside of occupational hazards [51].

Due to its volatility and solubility in water, ACN tends to either vaporize into the air or remain dissolved in water. They exhibit significant mobility in soil, potentially migrating to groundwater. While biodegradation is not anticipated to persist in either air or water, it might be hindered, especially at elevated concentrations in water. Research conducted by Phyllis A. et al. revealed that ACN biodegrades quickly to carbon dioxide at low concentrations. Surface soils are inhabited by many microorganisms that can break down acrylonitrile, allowing for quick and thorough degradation at low concentrations [52]. Data collected by the Environmental Protection Agency (EPA) from 2000 to 2022 in the National Air Quality System suggest a steady decrease in ACN in ambient air [53]. However, detectable concentrations of ACN in the atmosphere are usually linked to industrial origins. Nevertheless, there are currently no recent monitoring data available specifically around industrial areas. Additionally, data from the U.S. Water Quality Portal, including environmental monitoring data, reveal that no ACN was detected in surface waters across the U.S. The data on groundwater reveal a decrease in the ACN concentration from 2000 to 2022 [53].

ACN exposure in the general population primarily occurs through oral ingestion and inhalation. People commonly encounter this chemical through various products, such as acrylic fiber clothing, carpets, and plastics derived from acrylonitrile, which can potentially seep into food from plastic containers. The main sources of ACN release are underground injections and atmospheric emissions. It has been found at low levels in ambient air, groundwater, sediment, and surface water at production sites [53]. The main sources and environmental occurrence of ACN are summarized in Table 1 and Figure 1.

**Table 1:** Occurrences of toxins in soil and water ^1^.

Compound	Soil	Water
TCDD	Vietnam, Bien Hoa Airbase: 4.6–184.0 ppt [21] China, Tanggu District, Tianjin: 1.4 ppt [18] USA, New York, Niagara Falls: 10 ppt–670 ppm [15] USA, Missouri: 4.4–1750 ppb [15] Italy, Seveso: 81 ppb [54] USA, Missouri: 770–880 ppb [55] USA, Missouri: 1.9–723 ppb [56] USA, New Jersey, Newark: ~2300 ppb [57]	Russia, Ufa: 0.13–0.20 ng/L [27]
DEHP	Denmark (various locations): 1–3400 µg/kg dw [58] China, Guangdong, vegetable soil: 12.8–19.7 mg/kg [59] China, Zhejiang, proximity to e-waste recycling site: 0.2–14.1 mg/kg [60] China, Shandong, vegetable greenhouse: 1.9–25.2 mg/kg [61]	Germany, North Rhine, Westphalia: 0.33–97.8 mg/L [62] China, Songhua: 2.26–11.55 mg/L [63] Italy, Rieti District: 0.7–31.2 mg/L [64] Malaysia, Klang: 3.1–64.3 mg/L [65]
Bisphenol A	USA, Denver: 40–800 ng/g [66] China, urban spots: 166.0 ng/g (21 provinces) [67] Chennai, India: 200–17,400 ng/g (urban site) [44] Beijing: 380–1260 ng/g (urban site) [44] California Coast: 8–16 ng/g Bavaria, Germany: 5–15 ng/g (rural site) [68]	Japan, Tokyo Bay: 20.2–30.1 ng/L [69] Japan, Shizuoka, eight paper recycling plants: 8000–370,000 ng/L [70] USA, Bayou River: 9–44 ng/L [71] USA, max.: 420 ng/L (drinking water treatment plant waters) [72]

^1^ ACN is not typically present in the environment due to its volatility, despite being released through underground injections and atmospheric emissions, and is detected at low levels in air, water, and sediment near production sites.

### 3.2. Toxicity Caused by Toxins from Plastic and Plastic Waste

#### 3.2.1. Cytotoxicity

(a) TCDD

**Figure 2:**
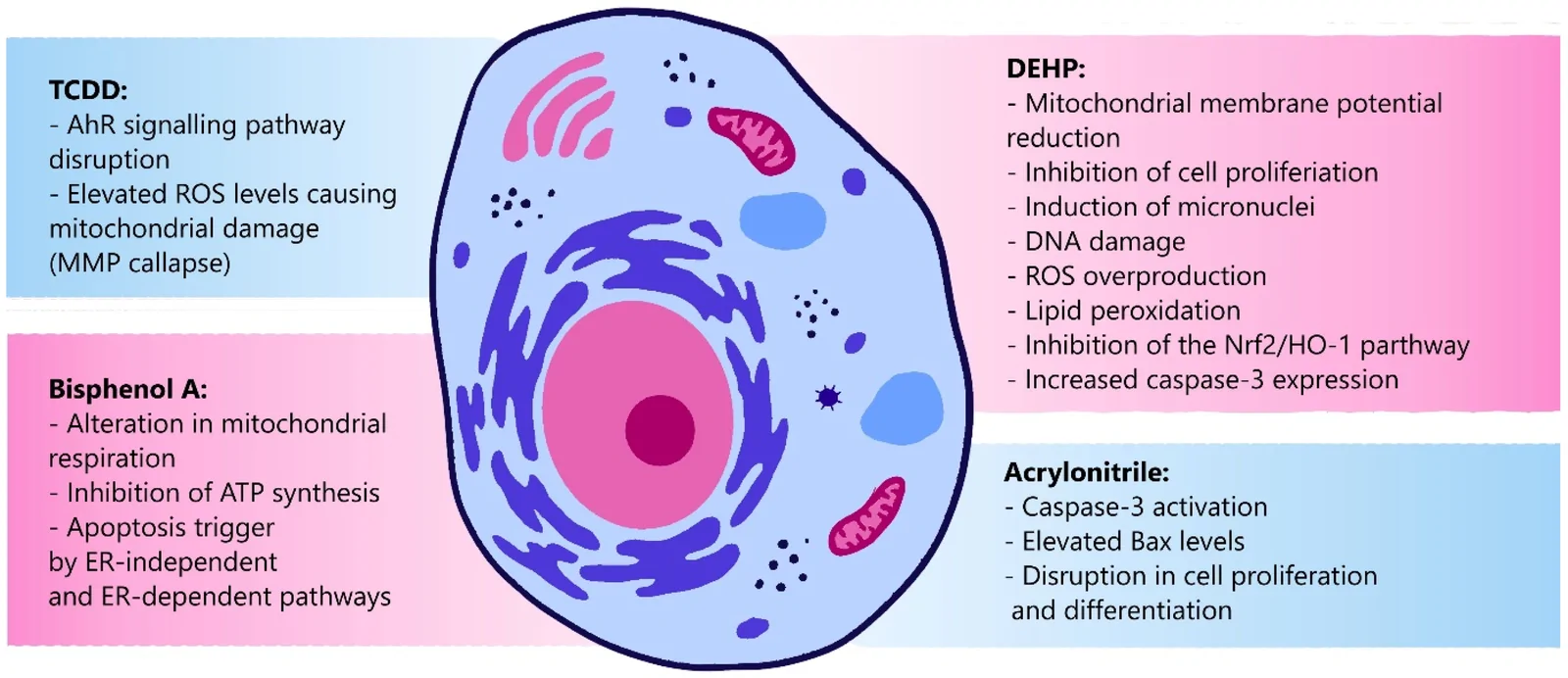
Cytotoxic effects caused by selected toxins.

Research has indicated that TCDD has significant cytotoxic effects, according to several studies. Suh et al. [73] reported that increased intracellular Ca^2+^ levels were essential factors triggering the death of MC3T3-E1 osteoblastic cells exposed to 100 nM TCDD. Their research focused on the role that oxidative stress induced by TCDD plays in causing mitochondrial damage and consequent apoptosis. Reactive oxygen species (ROS) are increased by TCDD exposure, which causes oxidative damage and structural abnormalities in the cell. This is accompanied by a significant decrease in the mitochondrial membrane potential, which is indicative of mitochondrial dysfunction and the initiation of the apoptotic cascade. Furthermore, increased production of nitric oxide (NO) and activation of genes encoding superoxide dismutase (SOD) point to a complex role for oxidative stress pathways. The cytotoxic effects are further enhanced by the activation of the extracellular signal-regulated kinase (ERK) signaling pathway and elevated nuclear factor kappa B (NF-kB) and ERK1 gene expression. TCDD-induced cytotoxicity is noteworthy because of its strong correlation with the aryl hydrocarbon receptor (AhR) pathway. Novelli and associates [74] investigated how TCDD affects the pancreatic islets of rats. After being exposed to different amounts of TCDD for six and twenty-four hours, significant increases in both cell necrosis and apoptosis were observed. Moreover, during longer exposure times, a clear relationship between dosage and increases in necrosis and apoptosis was found. The effects of TCDD on CD4 helper T cells, which are essential for adaptive immunological responses, were examined by Pang et al. [8]. At various TCDD concentrations (4 nM, 20 nM, and 40 nM), the immediate viability did not change, but over time, there was a noticeable decline in cell viability. Interestingly, TCDD exposure resulted in changes in inflammatory T helper (Th) cell subsets, particularly by increasing T helper 1 (Th1) cells and decreasing T helper 2 (Th2) and T helper 17 (Th17) cells, at both low and high doses (20 nM). Notably, lower doses suppressed inflammatory cell differentiation, whereas higher doses moderately decreased other cell types (Tregs). Following a 4-day culture period, high TCDD levels had a substantial effect on overall T-cell survival. These findings demonstrated that the constitutional differentiation of CD4 Th cell subsets is significantly influenced by the AhR signaling pathway. On the other hand, 48 h of exposure to 100, 200, or 500 nM TCDD did not result in appreciable decreases in cell viability, as shown by Yang et al. [75]. Their investigation revealed that Mouse Embryonic Stem Cells (mESCs) were not acutely cytotoxicated by the TCDD amounts that were evaluated. Research has shown that TCDD can interfere with the AhR signaling pathway in mouse embryonic stem cells (mESCs), which can increase the risk of malignant teratoma formation in vivo and increase the tumorigenic potential of ex vivo tumor cultures. Cytotoxic effects of TCDD are summarized in Figure 2.

(b) DEHP

A study conducted by Molino et al. [76] revealed a significant decrease in the viability of European sea bass embryonic cells (DLECs) after a 24-h incubation period with 0.01 mM DEHP. They verified that DEHP is known to cause necrosis, a process triggered by a transduction pathway that interacts with two different isotypes of the peroxisome proliferator-activated receptor (PPAR), PPARα and PPARβ. The results underscore the cytotoxic and genotoxic nature of DEHP in the DLEC embryonic cell line, prominently characterized by the inhibition of cell proliferation and the induction of micronuclei as primary effects. These findings suggest a potential aneugenic impact. Amara et al. [77] demonstrated the compound’s impact on human embryonic kidney 293 cells (HEK-293 cells), revealing a dose-dependent decrease in the metabolic ability of the cells to reduce the level of 3-(4,5-Dimethylthiazol-2-yl)-2,5-diphenyltetrazolium bromide (MTT), indicating its cytotoxic effects. The calculated half-maximal inhibitory concentration (IC_50_) after 24 h of cell exposure was approximately 400 μM. DEHP treatment induced intracellular ROS overproduction, disrupting the redox balance. SOD and (Catalase) CAT activities significantly increased, indicating an adaptive response. Elevated MDA (malondialdehyde) levels confirmed DEHP-induced lipid peroxidation. These findings revealed that Nrf2 and HO-1 expression decreased in a dose-dependent manner, suggesting that ROS inhibit the Nrf2/HO-1 pathway. DEHP also induces apoptosis by reducing the mitochondrial membrane potential and increasing caspase-3 expression. Lotfy et al. [78] explored the cytotoxicity of DEHP across human cancer cells, revealing its potent effect against human breast carcinoma (MCF7) with an IC_50_ of 6.941 µg/mL. It exhibited moderate cytotoxicity toward human liver carcinoma cells (HEPG2) with an IC50 of 26.73 µg/mL. However, its impact on human cervical carcinoma cells (HELA) was minimal, and no significant effect was observed on human colon carcinoma cells (HCT 116). In another study by Revathy et al. [79], the cytotoxic effect of DEHP on Oreochromis mossambicus, a freshwater fish, was investigated. Exposure to a 60 ppm concentration of this compound over 60 days induced the formation of micronuclei and various nuclear abnormalities, such as blebbing, notching, and lobing of nuclei, in a time-dependent manner. These assays highlighted a compromised metabolic capacity and DNA repair mechanisms in the fish due to DEHP exposure. DEHP induced a time-dependent increase in micronuclei formation and various nuclear abnormalities, including blebbing, notching, and lobing. These effects result in compromised metabolic capacity, reduced DNA repair efficiency, and a weakened defense mechanism against toxicants. Exposure to DEHP at a concentration of 29.1 μg/mL resulted in increased tail length and tail DNA percentage, indicating DNA damage. Cytotoxic effects of DEHP are summarized in Figure 2.

(c) Acrylonitrile

The cytotoxic capabilities of ACN are mostly related to its ability to cause high levels of oxidative stress. Research on human cell lines (neuroblastoma SH-SY5Y cells and human umbilical cord mesenchymal stem cells) demonstrated that ACN triggers apoptosis. ACN promotes the activation of caspase-3, a crucial enzyme associated with apoptosis, in a manner dependent on both the dose and duration of exposure. Furthermore, these studies revealed that ACN dose- and time-dependently elevated the levels of the proapoptotic protein Bax. The MTT assay revealed that incubation of cells with an ACN concentration of 1 µg/mL resulted in growth inhibition of approximately 50% [80]. Sun et al. [81] showed that ACN exerts cytotoxic effects on human umbilical cord-derived mesenchymal stem cells primarily through its metabolism to 2-cyanoethylene oxide, leading to the formation of DNA adducts. This disrupts cell proliferation and differentiation (particularly osteogenesis). Additionally, they reported that ACN treatment led to a dose-dependent decrease in the expression of cytokines involved in hematopoiesis, such as vascular endothelial growth factor, stem cell factor, and Fms-like tyrosine kinase 3 [81]. Research on JEG-3 and BeWo human choriocarcinoma cells revealed a decrease in cell proliferation upon ACN treatment for 48 h. A decrease in proliferation was accompanied by alterations in cell cycle regulatory proteins, with a decrease in cyclin D and cyclin E expression and an increase in the levels of p21 and p27, suggesting cell cycle arrest. Furthermore, analyses revealed that ACN treatment activated endoplasmic reticulum stress-related apoptosis pathways, as evidenced by increased expression of phosphorylated eukaryotic initiation factor 2 alpha protein, C/EBP homologous protein, and caspase 12. Exposure to ACN also modulated the expression of key proteins involved in regulating apoptosis. Specifically, p53 and Bax, proapoptotic markers, were upregulated, and B-cell lymphoma-extra large (BCL-XL l), an antiapoptotic marker, was downregulated. Additionally, studies have shown that ACN exposure of rat primary glial cells enhances lipid peroxidation and drastically decreases ATP levels [82]. Cytotoxic effects of ACN are summarized in Figure 2.

(d) Bisphenol A

BPA can interact with estrogen receptors and act as an antagonist via endocrine receptor (ER)-dependent signaling pathways. Research comparing the toxic effects of BPA on female and male amniocytes revealed that only ER-positive (female) amniocytes could not survive at concentrations of 40 and 100 μg/mL BPA. However, comparison of induced structural aberrations between ER-positive and ER-negative amniocytes at lower concentrations of BPA revealed no significant difference between these two cell groups [83]. These findings indicate that BPA has two pathways for inducing cytotoxicity: one that is ER-independent and one that is ER-dependent. Furthermore, a dramatic increase in cell death was detected only for ER-positive cells at higher concentrations. The ER-mediated pathway is more important for the cytotoxic effects of BPA. Investigation of HaCaT cells revealed that BPA inhibits growth and triggers apoptosis at concentrations greater than 20 μg/mL. Additionally, the results revealed a dose-dependent increase in the levels of the DNA damage markers Poly(ADP-ribose) polymerase (PARP) and caspase-3 in cells treated with BPA [84]. Nakagawa et al. researched freshly isolated rat hepatocytes and isolated hepatic mitochondria. In hepatocytes, exposure to BPA resulted in time-dependent cell death, with a decrease in intracellular ATP and total adenine nucleotide pools. BPA had concentration-dependent effects on mitochondrial respiration in isolated hepatic mitochondria. BPA inhibited oxygen consumption, particularly with NAD+-linked substrates and/or succinate, indicating impaired mitochondrial function. Notably, cytochrome oxidase-linked respiration remained unaffected by BPA [85]. In studies involving RAW264.7 macrophages conducted by Huang et al., BPA was found to induce cytotoxic effects in a concentration- and time-dependent manner. It triggers necrosis, apoptosis, and genotoxicity, with the underlying mechanisms involving mitochondrial disruption via downregulation of the anti-apoptotic proteins BCL2 and BCL-XL and upregulation of the proapoptotic proteins BAX, BID, and BAD. These alterations led to the phosphorylation of cytochrome C and p53. Both caspase-dependent pathways, including the cleavage of caspase-9, caspase-3, and PARP-1, and caspase-independent pathways, such as the nuclear translocation of apoptosis-inducing factors, are activated by BPA. Additionally, BPA induced the generation of ROS and reduced the activities of antioxidative enzymes [86]. Cytotoxic effects of TCDD, BPA, ACN and DEHP are summarized in Figure 2.

#### 3.2.2. Neurotoxicity

(a) TCDD

**Figure 3:**
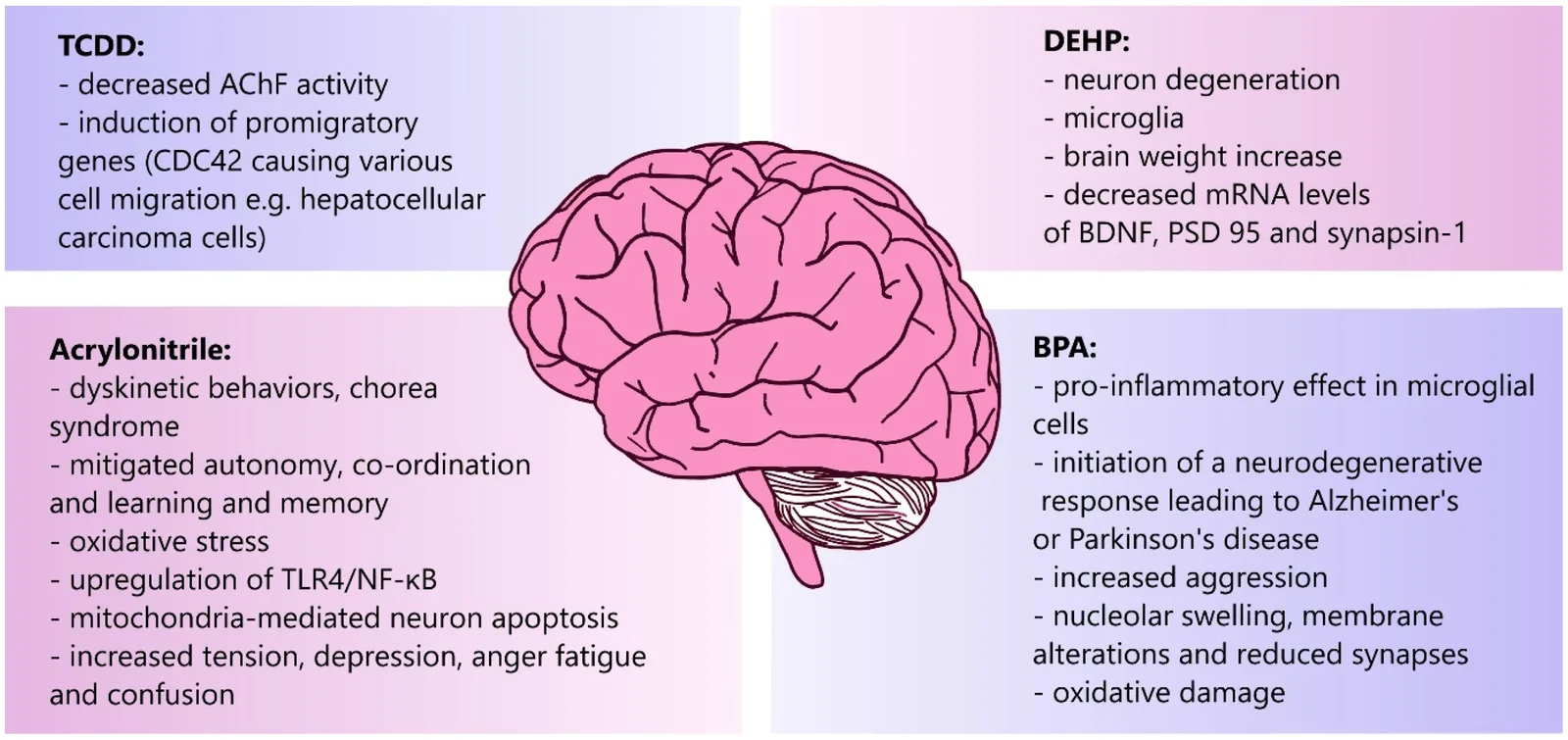
Neurotoxic effects caused by selected toxins.

The effects of TCDD on the enzymatic activity of acetylcholinesterase (AChE) in primary neurons at different developmental stages were examined by Sha et al. [87]. Their findings showed that AChE activity was reduced by TCDD treatment at 0.03 nM in comparison with the solvent control; the impact was more noticeable in immature neurons than in mature neurons. Interestingly, a nonclassical dose-dependent pattern developed, with a greater effect observed at lower TCDD concentrations. According to Chen et al. [88], relatively low-dose TCDD treatment (10^−10^ and/or 10^−9^ mol/L) can improve the capacity of primary astrocytes for horizontal and vertical migration. Primary astrocytes are the main type of cell in the central nervous system that is in charge of structural and homeostatic functions in a healthy brain. The biological evidence presented in their work suggests that dioxin promotes astrocyte reactivation by markedly upregulating the expression of promigratory genes. One of these genes, called CDC42 (cell division control protein 42 homolog), was identified as a mediator of cell migration. It is a member of the Rho-small GTPase family and an essential regulator of the actin cytoskeleton. Research has revealed that increased expression of CDC42 may promote cell migration in a variety of cell types, including those that cause hepatocellular carcinoma. Neurotoxic effects of TCDD are summarized in Figure 3.

(b) DEHP

In a study conducted by Yirun et al. [89], pregnant rats were given 30 mg/kg/day DEHP dissolved in corn oil. Several deteriorated neurons and microglia were observed in the brain tissue of the offspring after DEHP treatment, especially among the pyramidal neurons in the hippocampal CA1 area. There was also noticeable perivascular edema in this region. Condensed glial cells, neuron-free areas, and shrunken, degenerating neurons with darkly stained cytoplasm were found in the CA1, CA2, and CA3 regions of the hippocampal tissue. Compared with the control group, the present study revealed a 35% decrease in dopamine levels and a 47.4% decrease in dopamine transporter levels. Additionally, there was an 18% increase in relative brain weight. Qiu et al. [90] investigated how DEHP affects neurotoxicity-related pathways. Across all utilized doses (50 mM, 100 mM, 200 mM, and 400 mM), DEHP dramatically reduced the mRNA levels of brain-derived neurotrophic factor (BDNF), the synaptic protein PSD-95, and synapsin-1. In addition, it significantly decreased BDNF protein levels at all doses and synapsin-1 and postsynaptic density protein 95 (PSD-95) levels at concentrations of 100, 200, and 400 mM, indicating compromised neural synaptic function. In this study, DEHP treatment decreased the protein levels of p-Akt and p-PKA C at all doses and p-CAMK IV and p-CREB at concentrations of 100 mM, 200 mM, and 400 mM. Furthermore, DEHP (100 mM–400 mM) downregulated PSD-95 and synapsin-1 proteins in N2a cells, whereas synapsin-1 levels slightly increased at 50 mM DEHP. The ability of the cells to withstand and adjust to external stress caused by DEHP exposure was demonstrated by this stimulatory effect at a relatively low exposure concentration (50 mM), indicating a potential tolerance and homeostatic mechanism activated by DEHP exposure. Neurotoxic effects of DEHP are summarized in Figure 3.

(c) Acrylonitrile

In experimental studies on rats, exposure to ACN provided distinct insights into the neurotoxic effects of ACN. Rats exposed to ACN at levels of 15 mg/kg/day or higher exhibited dyskinetic behaviors, circling, and chorea syndrome. ACN exposure in rats depleted glutathione in various brain regions, indicating oxidative stress [91]. Another study of ACN-induced neurotoxicity in rats revealed mitigated autonomic activities, motor coordination, and learning and memory, decreased neuronal morphology, and increased oxidative stress after ingesting 46 mg/kg of ACN daily. ACN upregulated the TLR4/NF-κB signaling pathway, increased the expression of inflammatory markers (IL-6 and TNF-α), and induced mitochondria-mediated neuronal apoptosis [92]. Personnel who work in environments where ACN is present have demonstrated signs of cognitive impairment, as indicated by a thorough battery of neurobehavioral core tests recommended by the World Health Organization (WHO). With respect to the profile of mood states, employees in ACN-related plants reported higher levels of stress, despair, rage, exhaustion, and confusion. Additionally, these exposed people performed worse on neurobehavioral measures, such as digit span, Benton Visual Retention Test, Pursuit Aiming II, and simple reaction time. The cumulative effect of ACN on human cognitive and mood-related processes was highlighted by the correlation between the severity of these impairments and the length of exposure [93]. Neurotoxic effects of ACN are summarized in Figure 3.

(d) Bisphenol A

Many studies have examined the effects of exposure to low doses of BPA during critical periods of brain development on subsequent brain structure, function, and behavior. Research conducted on BPA released from plastic bottles into the water has shown that it has neurotoxic effects on developing cerebellar neurons [94]. Additionally, the transcription factor NF-κB can be activated by BPA. These findings indicate that BPA has a proinflammatory role in microglia and that it might initiate a neurodegenerative response that progresses to Alzheimer’s disease or Parkinson’s disease [95]. Sprague–Dawley rat offspring prenatally exposed to BPA (administered orally to mothers throughout gestation) at a dose of 4 ng/kg/day presented increased aggression. In addition, increased aggressiveness (using a composite score of aggression) in male CD-1 mouse offspring occurred as a result of the oral administration of 2 ng/(kg day) of BPA to pregnant females [96]. Zhihong et al. reported the neurotoxic effects of BPA on Neuro-2a cells. Ultrastructural and morphological analyses revealed significant cellular changes, including nucleolar swelling, nuclear membrane alterations, and reduced synapses. Overall, these findings indicate that BPA disrupts the development and proliferation of Neuro-2a cells by damaging their cellular and synaptic integrity and causing cytoskeletal injury [97]. The findings also revealed that minimal exposure to BPA might cause oxidative damage and neurotoxic effects in aquatic organisms. Research on zebrafish suggests that exposure to a concentration of 1500 ng/L BPA results in a decrease in the overall distance covered and an increase in the duration of freezing [98]. Neurotoxic effects of BPA are summarized in Figure 3.

#### 3.2.3. Cardiotoxicity

(a) TCDD

**Figure 4:**
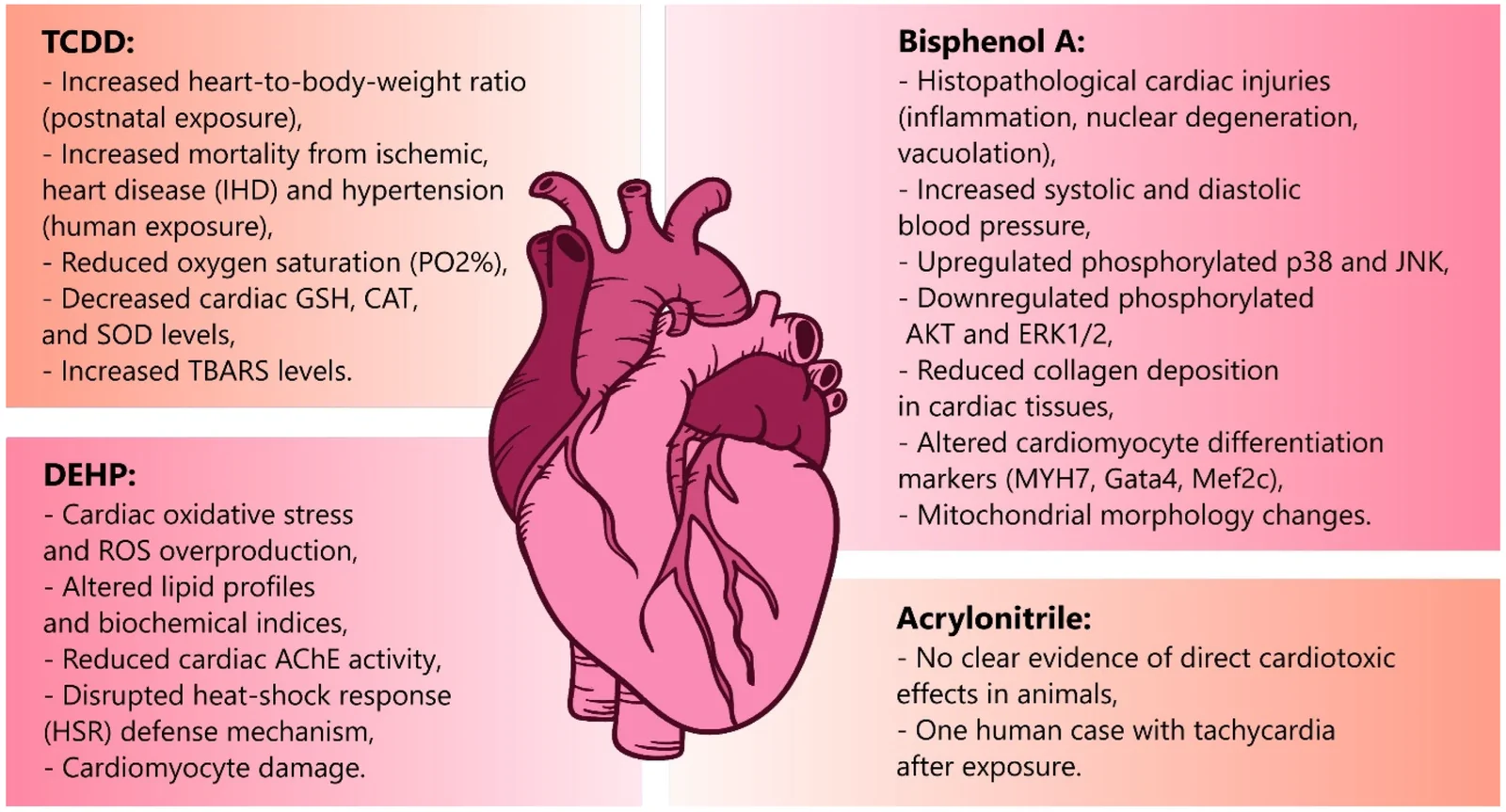
Cardiotoxic effects caused by selected toxins.

Fujisawa et al. [99] confirmed that administering TCDD (80 µg/kg) to dams during the postnatal period increases the heart-to-body-weight ratio. Their research revealed a risk posed to the heart during both the pre- and postnatal periods, revealing seemingly contrasting effects of TCDD on heart-to-body-weight ratios depending on developmental stage. Notably, postnatal TCDD exposure led to an increase in heart-to-body weight accompanied by decreased body weight, whereas prenatal exposure did not induce the same effect. In 1976, an herbicide explosion in Seveso, Italy, resulted in significant TCDD exposure among residents. In 2008, Consonni et al. [100] reported increased mortality rates from ischemic heart disease (IHD) among male residents and increased mortality from hypertension among female residents. These findings underscore the potential of TCDD to induce cardiotoxicity during development. Ediz et al. [101] investigated the effects of TCDD exposure on rat hearts and the protective role of melatonin. Orally administering TCDD at a dosage of 2 mg/kg/week for 45 days led to impaired body weight, heart weight, hemodynamic parameters (arterial pressure and blood pressure), and oxygen saturation (PO2%) in the rats. This reduction in oxygen saturation may be related to inadequate pulmonary blood flow and progressive decreases in PO2 levels that occur with the onset of heart failure. Furthermore, TCDD treatment resulted in a significant decrease in the cardiac levels of glutathione (GSH), CAT, and SOD but an increase in thiobarbituric acid reactive substances (TBARS). Although there is evidence that exposure to TCDD in humans is associated with cardiovascular illnesses, there is still a lack of animal models that fully explain the underlying mechanisms causing these effects. Cardiotoxic effects caused by TCDD are shown in Figure 4.

(b) DEHP

In Wang et al.’s [102] study, exposure to DEHP at 750 mg/kg BW/d resulted in heart injury in quail. Heat shock transcription factors (HSFs) and heat shock proteins (HSPs) are components of the heat shock response (HSR) defense mechanism, which is activated as a result of this exposure. Elevated DEHP concentrations increased the anti-stress potential of the HSR, controlling HSPs by increasing HSF expression and ultimately providing cardioprotective benefits. However, high DEHP concentrations overtaxed the HSR defense mechanism, disrupting the expression of certain HSPs and ultimately wiping out their anti-stress capacity. Amara et al. [103] investigated the effects of daily DEHP intake (at doses of 5, 50, and 200 mg/kg over 30 days) on mouse hearts. The lipid profiles and biochemical indices of DEHP-treated mice were altered, which may have contributed to cardiac damage in these animals. Moreover, cardiac AChE activity was reduced, and oxidative stress was increased by DEHP exposure. The production of ROS, which alter several cardiomyocyte components, is likely the cause of the harmful effects of DEHP. Cardiotoxic effects caused by DEHP are shown in Figure 4.

(c) Bisphenol A

Mahmoud et al. researched the cardiotoxic effects of 50 mg/kg BPA per day on rats. BPA significantly induced histopathological injuries, including focal lymphatic inflammation, nuclear degenerative changes, cytoplasmic vacuolation, and changes in systolic and diastolic blood pressure. Furthermore, they reported that BPA upregulated phosphorylated p38 and c-Jun N-terminal kinase but downregulated phosphorylated AKT (protein kinase B) and ERK1/2 (extracellular signal-regulated protein kinases 1 and 2) [104]. Additionally, BPA reduces the deposition of collagen in rat cardiac tissues. BPA significantly stimulated the proliferation and migration of cultured cardiac fibroblasts, as well as collagen production. Furthermore, BPA exposure enhances the phosphorylation of ERK1/2 [105]. Shoufei et al. studied embryonic stem cells chronically exposed to BPA. They reported a reduction in the expression of cardiomyocyte differentiation markers, such as *MYH7*, *GATA4*, and *MEF2C*. Furthermore, BPA exposure induced changes in mitochondrial morphology and the ratio of microfilaments to mitochondrial length. Additionally, bisphenol AF (BPAF) exposure influences the expression of G protein-coupled estrogen receptors, with concentration-dependent changes observed at both the mRNA and protein levels [106]. Cardiotoxic effects caused by BPA are shown in Figure 4.

(d) Acrylonitrile

No studies have investigated the cardiovascular effects in animals following inhalation exposure. Intermediate- and chronic-duration inhalation and oral studies have reported no cardiovascular effects in laboratory animals, except for increases in heart weight. Since there is no other evidence of heart damage, weight increases are not considered adverse effects of acrylonitrile exposure. In the absence of other evidence of cardiac damage, these increases were not considered adverse effects of acrylonitrile exposure [53]. In humans, limited evidence is available from individual acute exposure cases. One reported case involved a 3-year-old child who slept in a room fumigated with acrylonitrile. Tachycardia, respiratory dysfunction, and lip cyanosis were among the symptoms reported prior to death. Although this case provides evidence of cardiovascular symptoms following acute acrylonitrile exposure, the available information is insufficient to establish a causal relationship between acrylonitrile exposure and cardiac injury. Therefore, this case is considered complementary human evidence, rather than conclusive evidence of cardiovascular toxicity, as shown in Figure 4 [107].

#### 3.2.4. Reproductive Toxicity

(a) TCDD

**Figure 5:**
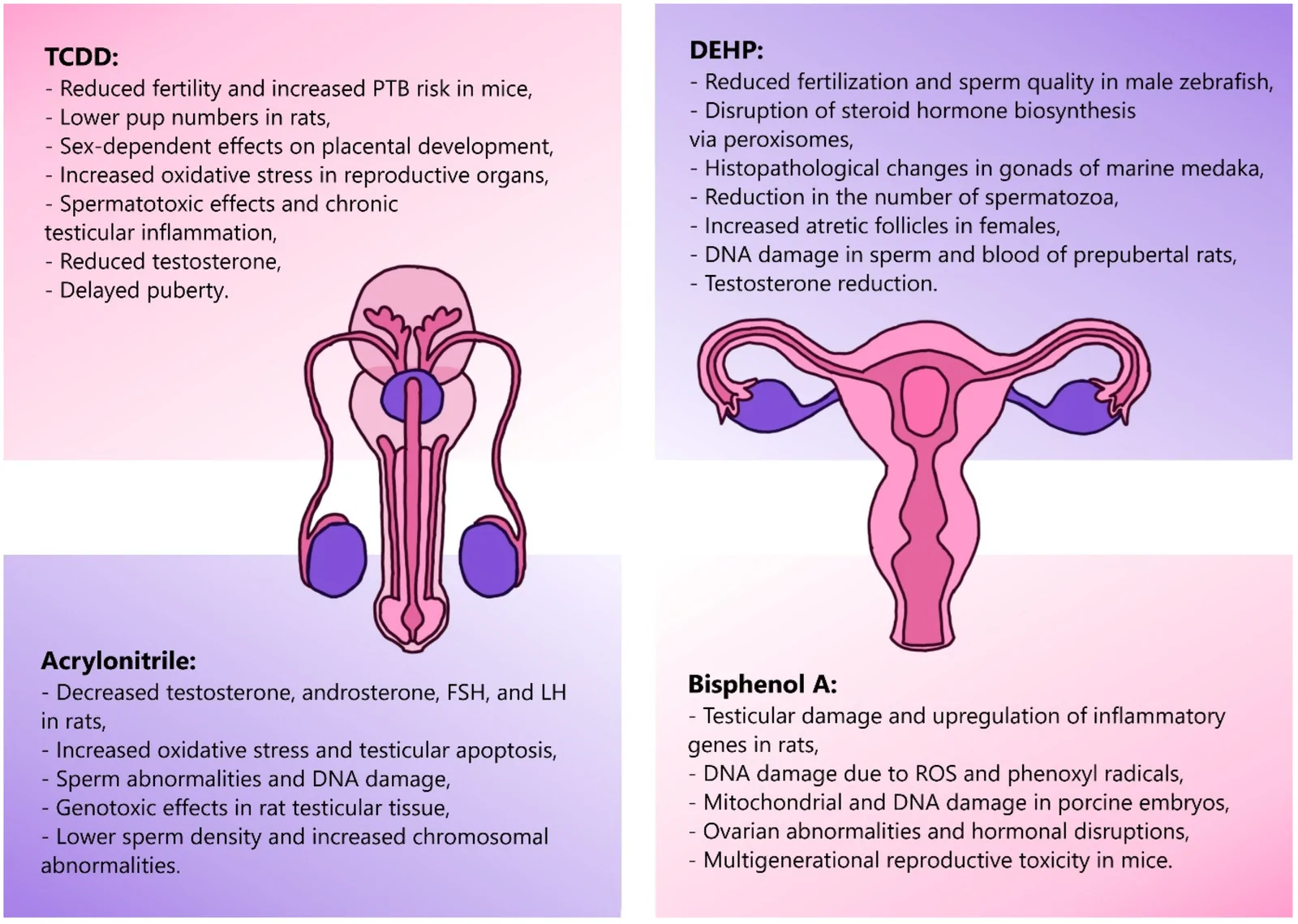
Reproductive toxic effects caused by selected toxins.

Pregnant rodents exposed to TCDD exhibited transgenerational effects on pregnancy outcomes, including reduced fertility and increased risk of spontaneous preterm birth (PTB) in F3 mice [108]. Ancestral TCDD exposure in F3 Sprague–Dawley rats led to lower pup numbers [109], in contrast with more recent findings [110]. TCDD exposure in utero increased infertility and PTB rates in F1 mice with latent mouse parvovirus infection [108]. Developmental TCDD exposure increased sensitivity to inflammatory challenges across generations. Maternal and paternal TCDD exposure in mice affects group B Streptococcus (GBS) infection differently based on sex, revealing sex-dependent differences in placental development and infection response after early-life TCDD exposure. Ancestral TCDD exposure resulted in intrauterine growth restriction (IUGR) and smaller placentae in F3 males [111] and lower body weight in F3 dams [112]. However, no sex ratio differences were observed [113]. Male reproduction studies have shown inconsistent transgenerational changes in sperm concentration and motility [114], with varying impacts on sperm morphology [112,115]. Mocarelli et al. investigated residents of Seveso, Italy, who were exposed to TCDD (mean peak exposure = 32 pg/kg b.w. per day). In men exposed during infancy or prepuberty, TCDD was associated with reduced sperm concentration and progressive motility, as well as altered reproductive hormone levels, including decreased estradiol and increased FSH. Interestingly, exposure during puberty was associated with increased total and motile sperm counts, while adult exposure showed no significant effects on semen quality. The findings suggest that the timing of TCDD exposure is critical and that early-life exposure may cause persistent alterations potentially contributing to declining male fertility [116]. TCDD-exposed mice presented increased spermatotoxic effects, altered sperm morphology, chronic testis inflammation, reduced sperm quality, and infertility. F3 males also showed reduced fertility and increased PTB rates [115], while testosterone reduction was observed by Skinner’s group [114], and fewer implanted embryos were found [112]. Additionally, F3 animals showed a drastic reduction in testosterone levels without changes in luteinizing hormone (LH) levels [114], possibly due to chronic inflammation [108]. Puberty onset investigations revealed abnormalities in 40% of F1 males and delayed puberty in 5% of ancestrally exposed F3 males [110]. Animal studies underscore the significant impact of TCDD on both the female and male reproductive systems, which affects fetal development through puberty. TCDD disrupts sex hormone signaling, interacts with steroid receptors, and modifies steroid biosynthesis, which is critical for sexual differentiation, fertility, and reproductive behavior [114,117,118]. TCDD also decreases antioxidant enzyme activity, increases ROS generation in reproductive organs [119,120], and alters the protamine/histone ratio, contributing to DNA oxidative damage [121]. Reproductive effects caused by TCDD are summarized in Figure 5.

(b) DEHP

Ma et al. [122] tested the sex-dependent toxicological effects of DEHP in male zebrafish and reported that chronic exposure to DEHP (100 μg/L) reduces egg fertilization, spermatogenesis, and sperm maturation in male zebrafish. Transcriptomic profiles and in silico modeling suggested the involvement of peroxisomes, PPAR signaling, and steroid hormone biosynthesis pathways in the reproductive toxicity of DEHP in males. DEHP treatment significantly inhibited spermatogenesis, affecting sperm quality in males and reducing the fertility rate. The effect of DEHP on peroxisomes interfered with steroid hormone biosynthesis, affecting male reproductive behavior. All of the peroxisome-related genes (pex6, pex16, hacl1, gnapt, pex11a, pex11b, and nudt7) were upregulated in the DEHP exposure groups, indicating that an impact on androgen and estrogen balance is crucial for spermatogenesis and sperm maturation. Christiansen et al. [123] examined the effects of perinatal DEHP exposure on male reproductive development in Wistar rats. Even at a relatively low dose of 10 mg/kg/day, DEHP reduced anogenital distance, increased nipple retention, decreased the weight of the levator ani/bulbocavernosus muscles and prostate, and caused mild genital abnormalities. Higher doses additionally induced testicular histopathological changes, reduced testicular weight, and altered the expression of androgen-regulated genes in the prostate. These findings demonstrate that DEHP can affect male reproductive development at low doses and raise concerns regarding the potential reproductive effects of perinatal DEHP exposure in humans. Ye et al. [124] exposed marine medakas (*Oryzias melastigma*) to DEHP, which accelerated spawning start time and decreased fecundity in females paired with unexposed males. This also resulted in a reduction in the fertilization success of oocytes spawned by untreated females. Research has shown that DEHP exposure induces sex- and tissue-specific effects on the expression of sex steroid hormone receptors and steroidogenesis-related genes in the gonads and brains of male and female medakas. They discovered that DEHP exposure led to histopathological changes in the gonads of both male and female medakas. A reduction in the number of spermatozoa, increased atretic follicles in females, and altered gonadal morphology were observed. Karabulut and Barlas [125] investigated the influence of DEHP on male rats starting in the prepubertal period and concluding at puberty, demonstrating direct DNA damage in blood and sperm, along with detrimental effects on the reproductive system in rats spanning the prepubertal to pubertal period. Their research revealed elevated levels of DNA damage in blood lymphocytes and sperm, with significant increases in tail intensity. Testosterone levels were reduced, and DEHP led to a significant decrease in leukocyte levels and hemoglobin content and an increase in the mean cell volume (MCV) (400 mg/kg per day). Reproductive effects caused by DEHP are summarized in Figure 5.

(c) Acrylonitrile

Daily oral administration of ACN resulted in decreased serum testosterone, androsterone, follicle-stimulating hormone (FSH), and LH in albino rats and Sprague–Dawley rats. ACN-induced injuries include diminished GSH, increased MDA, and severe alterations in seminiferous tubules [126]. Moreover, investigations of rats exposed to ACN demonstrated that testicular apoptosis was caused by oxidative stress. Testicular apoptosis is facilitated by the activation of nuclear factor-κB signaling by ACN. Male mice exposed to ACN had lower-quality sperm. Sperm abnormalities and DNA strand breakage are caused by ACN [92]. Covalent binding to DNA was shown by the in vivo contact of acrylonitrile with rat testicular tissue, which affected DNA synthesis and repair. According to previous studies, acrylonitrile interferes with DNA activity in testicular tissue, acting as a genotoxic agent and possibly disrupting male reproductive function [127]. The sperm density and number per ejaculate in the exposed group were significantly lower than those in the control group, according to an investigation into the effects of ACN exposure on semen quality and genetic abnormalities in the spermatozoa of ACN-exposed workers. In addition, the exposed group presented longer mean tail lengths and greater numbers of comet sperm nuclei, both of which are signs of DNA damage. In addition, the exposed group had a significantly greater frequency of sex chromosomal disomy than the control group did, especially for XY-bearing sperm [127]. Reproductive effects caused by ACN are summarized in Figure 5.

(d) Bisphenol A

BPA-induced reproductive toxicity has been reported to be attributed mainly to oxidative stress. Research on male albino rats exposed to BPA for 70 days revealed testicular damage. Moreover, marked DNA internucleosomal fragmentation patterns, as well as the upregulation of cyclooxygenase-2 and estrogen receptor-2 NSe genes, were detected [128]. BPA also induces damage to DNA through phenoxyl radicals and ROS production, which can induce direct DNA alterations amplified by the impairment of antioxidant enzyme pathways. Increased ROS production and lipid peroxidation were observed when *Drosophila melanogaster* was exposed to BPA in food. Moreover, BPA treatment of porcine embryos increased ROS formation, causing cytochrome c release, mitochondrial and DNA damage, and apoptosis through the p53–p21 signaling pathway. Following ex vivo exposure of rat ovaries to BPA [129]. Additionally, K. Barry Delclos et al. [130] reported that high doses of BPA (300,000 μg kg bw/day) given to rats reduce gestational growth, alter ovarian health (such as heightened cystic follicles, diminished corpora lutea, and antral follicles), and disrupt serum hormone levels (with elevated serum estradiol and prolactin and decreased progesterone). Polash et al. [131] studied the effects of BPA exposure on the reproductive systems of adult male mice and their offspring. After six weeks of BPA exposure, male mice were allowed to reproduce, producing the F1–F4 generations. The findings demonstrated that testicular abnormalities and changes in germ cell proportions continued in generations F0–F2, with a lower spermatogonial count in F0 alone, even at the LOAEL. These results imply that adult male BPA exposure may affect the reproductive health of future generations. Reproductive effects caused by BPA are summarized in Figure 5.

#### 3.2.5. Immunotoxicity

(a) TCDD

**Figure 6:**
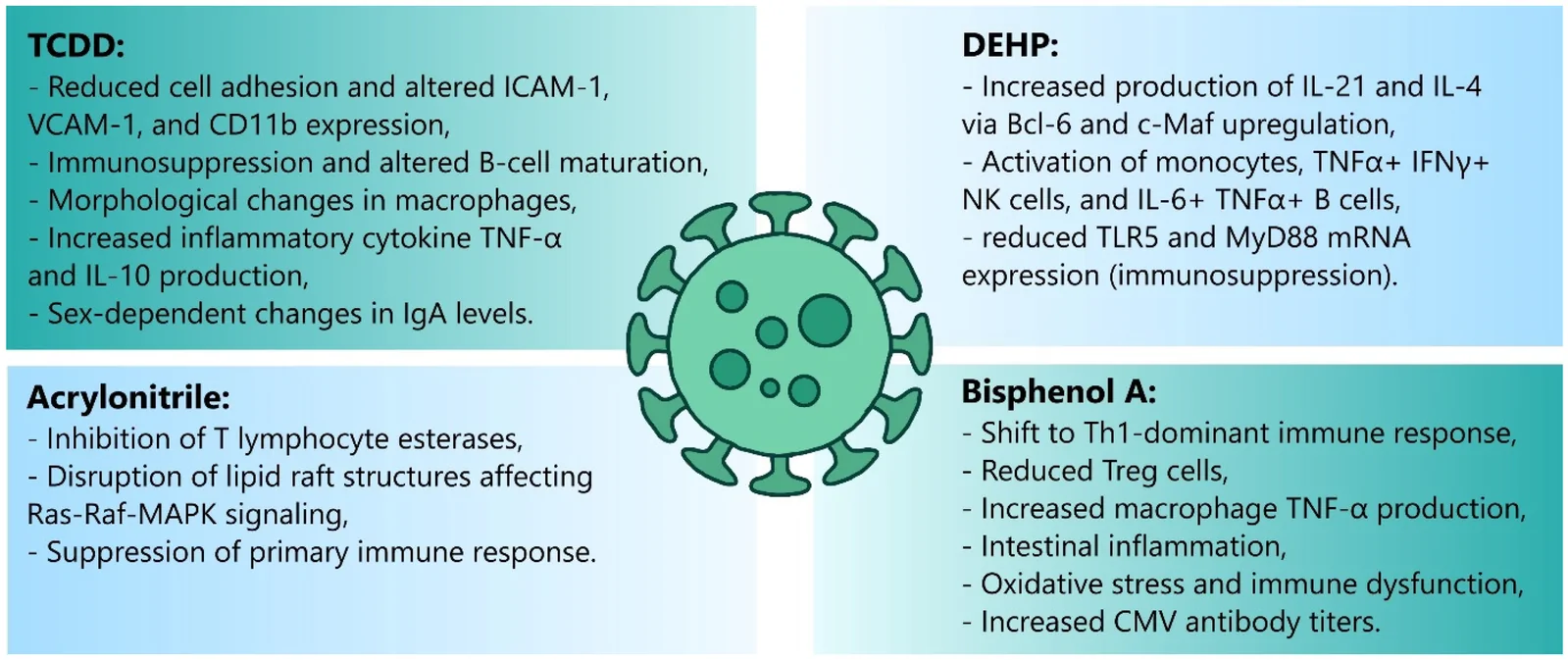
Immunotoxic effects caused by selected toxins.

Li et al. [132] investigated the impact of exposure to TCDD, including environmentally relevant concentrations, on immune functions in THP-1-derived macrophages. Cell adhesion, adhesion molecules, morphology, and cytokine/chemokine production were evaluated via high-throughput techniques. The results revealed that when TCDD was administered, there was a notable decrease in cell adhesion and changes in the expression of adhesion molecules (ICAM-1, VCAM-1, and CD11b). Morphological alterations, including a decrease in the number of pseudopodia and the expression of F-actin, were noted. Through the generation of TNF-α and IL-10, TCDD increases inflammation by influencing the production of several cytokines/chemokines. Important hub genes (TNF, SRC, FGF2, PTGS2, CDH2, GNG11, BDNF, WNT5A, CXCR5, and RUNX2) linked to TCDD-induced immunotoxicity were discovered via genomic research. Feng et al. [133] presented the effects of TCDD exposure on humans in an effort to understand the mechanisms underlying the elevated incidence of lymphoma and lowered humoral immunity linked to long-term TCDD exposure. They replicated human exposure outcomes by observing an overall immunosuppressive impact following 20 weeks of TCDD exposure in a mouse model. In splenic B cells, transcriptome analysis revealed a molecular signature suggestive of lymphogenic transdifferentiation and delayed maturation. Foxx et al. [133] researched diurnal variations in fecal sIgA levels in mice caused by TCDD. The serum IgA and fecal sIgA concentrations changed in a dose- and sex-dependent manner following exposure, suggesting a possible effect on mucosal immunity. The results of the present study revealed a complicated link between sIgA levels and AhR activation, with both improving and suppressive effects observed at different dose ranges. Furthermore, there were differences in sensitivity to AhR-mediated immune modulation based on sex. Immunotoxicity caused by TCDD is summarized in Figure 6.

(b) DEHP

Han et al. [134] demonstrated that DEHP exerts adjuvant toxic effects on Tfh cells, inducing excessive synthesis of the cytokines IL-21 and IL-4 through the upregulation of the transcription factors Bcl-6 and c-Maf. Consequently, there is increased secretion of allergy-related Immunoglobulin E and Immunoglobulin G1. Nygaard et al. [135] reported that exposure to DEHP may activate specific innate immune cell subsets while suppressing certain adaptive immune cells. Notably, monocytes, TNF-α+ IFNγ+ CD11c+ activated NK cells, and IL-6+ TNF-α+ B cells were consistently different between the high and low DEHP exposure groups. Yuan et al. [136] reported that the exposure of yellow catfish to DEHP had no effect on survival but significantly improved growth performance and feed utilization. Oxidative stress was indicated by an increase in the MDA concentration and an inhibition of antioxidant enzyme activity. The phagocytic indices decreased, whereas the immune response indicators, complement levels, total immunoglobulins, and respiratory bursts did not change. TLR5 and MyD88 mRNA expression decreased, indicating immunosuppression. Immunotoxicity caused by DEHP is summarized in Figure 6.

(c) Acrylonitrile

Epidemiological investigations and animal studies have already established that ACN affects lymphocytes and spleen. The primary immunotoxic mechanism of ACN involves the inhibition of T lymphocyte esterases and the α (3) component of cytochrome c oxidase among immunocyte mitochondrial respiration enzymes [137]. Additionally, studies have suggested that ACN causes immunotoxicity by damaging lipid raft structures, leading to the separation of the Bcl10 protein (which is crucial in lymphocyte development) and lipid rafts, ultimately affecting the Ras–Raf–MAPK–ERK signaling pathways [138]. ACN induces immunotoxicity through its anticholinesterase activity and general toxicity, which inhibits components of the immunocyte respiratory enzyme system, particularly hydrogen cyanide. Collectively, these effects affect primary and secondary immune responses and cause immunosuppression. The suppression of the primary immune response by ACN is evident in the significant inhibition of delayed-type hypersensitivity reactions and a reduction in the number of antibody-producing cells in the spleen. These findings indicate that the body’s capacity to develop a successful immunological response in response to antigen exposure is compromised. Since esterases are primarily found in T lymphocytes, AN’s anticholinesterase activity helps explain why it has an immunotoxic effect on these cells. Furthermore, immunosuppression occurs in a variety of immunocyte populations as a result of the reduction in immunocyte respiration caused by AN, which is likely caused by the emission of hydrogen cyanide [139]. Immunotoxicity caused by ACN is summarized in Figure 6.

(d) Bisphenol A

At a more immunological level, BPA can change the Th1/Th2 ratio by altering T-cell proliferation. Most studies have concluded that the immune response becomes Th1-dominated by a decrease in Treg cells and an increase in the production of TNF-α and nitric oxide by macrophages [140]. BPA promotes intestinal inflammation, increasing the number of infiltrating immune cells in the colon and increasing the expression of Ghsr and proinflammatory cytokines and chemokines, such as Il6 and Ccl2, in the colonic mucosa [96]. Various irregularities, such as heightened oxidative stress, compromised innate and adaptive immune functions, and increased levels of insulin and blood glucose, can be identified following exposure to BPA. Research has shown that these abnormalities are caused by the ability of BPA to bind to the STAT3 and IL10 genes, inducing immunotoxicity [141]. Furthermore, high urinary levels of BPA in humans were significantly correlated with antibody titers to cytomegalovirus (CMV). Increased CMV antibody titers indicate a stressed immune system and can be an early marker of immune dysfunction in humans [142]. Immunotoxicity caused by BPA is summarized in Figure 6.

### 3.3. Heat Shock Proteins (HSPs) as a Biomarker of Cellular Stress Response Induced by Toxins

HSPs are a family of stress-responsive proteins, which includes most of the molecular chaperones. The main role of molecular chaperones is cell protection from physiological and environmental insults such as hyperthermia, ischemia, anoxia, toxins, UV light, viral particles, or other types of environmental stressors. These biologically active particles protect cellular proteins from degradation, oxidative stress, hypoxia, and thermal stress. Moreover, HSPs, which possess molecular chaperone activity, are essential during stress-free periods in various important housekeeping functions such as protein folding and maturation, maintenance of proteome integrity, and protein homeostasis (proteostasis) [143,144,145].

Research on cellular oxidative stress (OS) has shown that the expression patterns of HSPs are the major cellular biomarkers of cellular responses against stressors, such as heat stress, oxidants, toxins, free radicals, viruses, heavy metal exposure, pesticide exposure, and hypoxia. HSPs are also called the “stress proteins” [146,147]. In the study by Mitra et al. (2018), water quality characteristics were measured. The authors of the study sought existing correlations between water quality parameters, dissolved oxygen (DO), biochemical oxygen demand (BOD), chemical oxygen demand (COD), pH, conductivity, and changes inHSP gene expression [146]. The experiments detected a correlation between the expression patterns of HSP47 and HSP70b and BOD, which is an indicator of organic pollution. It was found that the upregulation of HSP47 and HSP70b correlated well with POP concentrations (aldrin and HCH). Aldrin and HCH were responsible for HSP upregulation, indicating a synergistic effect of toxins on the upregulation of HSP47 and HSP70b as a biomarker response [146].

OS induced by hazardous compounds can affect the activity of the network of global regulatory genes (e.g., the guardian of the genome—p53 [144] or grpE [148]) and disrupt the proper expression of the organized network of HSP genes and genome stability, which can lead to the induction of carcinogenesis. It is like hitting a ball into the goal, and the entire goal vibrates. In the “ball and goal” theory, the ball is persistent organic pollutants (POPs), which “hit” the properly functioning network of global regulatory genes and HSP genes in the cell, leading to a loss of homeostasis and genome stability. This state can lead the cell to a pathway of cancerogenesis. Moreover, toxins from plastics acting on healthy cells may affect the expression and activity of microRNA (miRNA); these chemicals can also induce protooncogenes or block the expression of suppressor genes (e.g., p53) and DNA damage repair system genes, which may lead to the induction of carcinogenesis.

### 3.4. The Implication of Persistent Organic Pollutants in Cancerogenesis

Previous scientific studies have demonstrated a link between human exposure to POPs and increased incidence and aggressiveness of various cancers [149,150]. Arfin et al. (2021) and Azizi et al. (2022) [151,152] review the current state of knowledge and argue that the main mode of action involved in POP-inducible risk of cancer progression is OS. Cell exposure to POPs induces cell damage and stress caused by OS, leading to an imbalance between cellular antioxidant mechanisms and reactive nitrogen species (RNSs) and ROSs. OS conditions induce multiple mechanisms, such as DNA damage, inflammation, and angiogenesis, which increase cancerogenesis [151,152].

Environmental and dietary exposure to POPs may induce the development of colorectal cancer. To date, it has been established that the induction of colorectal cancer involves a combination of genetic and epigenetic mechanisms [153,154]. Numerous laboratory experiments have demonstrated that POPs can affect genome stability and function through epigenetic mechanisms such as DNA methylation, histone modifications, N6-methyladenosine (m^6^A) modification, and microRNA (miRNA) expression [155].

### 3.5. Summary and Future Prospects

Plastic-derived toxins represent a unique intersection of material science, toxicology, and environmental health. The widespread use of plastic-based materials in industrial processes and consumer products has contributed to the release and distribution of associated toxic substances across ecosystems and their accumulation in living organisms. Despite structural differences and varied functional roles, these toxins can exhibit convergent toxicodynamic mechanisms. The discussion of their multifaceted effects highlights the complex and systemic nature of toxicity associated with plastic-derived substances. The cytotoxicity of these substances is primarily mediated through mechanisms involving oxidative stress, mitochondrial dysfunction, and impaired cellular signaling, with TCDD and BPA showing particularly strong apoptotic and genotoxic profiles. DEHP and ACN, which are commonly associated with plastic materials and industrial emissions, contribute to DNA damage, impaired metabolic function, and altered redox homeostasis in various cell types. BPA, derived primarily from polycarbonate plastics and epoxy resins, exhibits both receptor-dependent and receptor-independent pathways of toxicity, affecting cell viability and endocrine function. Importantly, even low-dose exposure to these substances, often within environmentally relevant concentrations, is sufficient to elicit measurable toxicological responses. A striking observation from this review is the dose- and time-dependent toxicity of these plastic-associated toxins, with adverse effects often manifesting even at environmentally relevant concentrations. For example, TCDD exposure in THP-1-derived macrophages altered adhesion molecule expression and reduced pseudopodia formation at concentrations relevant to real-world contamination scenarios [128], whereas long-term low-dose exposure in mice induced immunosuppressive molecular signatures in splenic B cells [133]. DEHP displayed a biphasic response in the heat-shock response pathway of quail hearts, where low to moderate doses upregulated protective HSP expression, but chronic high doses overwhelmed this defense, leading to cardiac injury. Similarly, BPA elicited histopathological and signaling pathway alterations in rat cardiac tissue at doses far below acute toxicity thresholds [102] and induced proinflammatory gene expression in the colonic mucosa following environmentally plausible exposure [97]. The immunotoxicity of acetonitrile also follows this pattern, with evidence that relatively low-level, sustained exposure impairs delayed-type hypersensitivity responses and reduces the number of antibody-producing cells [139]. Such examples illustrate that regulatory approaches relying solely on high-dose hazard thresholds may underestimate the true ecological and public health risks, as chronic low-dose exposure to plastic-associated toxins can elicit subtle but biologically significant changes in multiple organ systems. The stability and potential for bioaccumulation of these substances increase their ecological footprint, with implications for wildlife health and ecosystem integrity. Aquatic species, in particular, demonstrate vulnerability to even trace levels of plastic-associated toxins, resulting in developmental, behavioral, and reproductive abnormalities. These effects may compromise biodiversity and ecological stability, with potential consequences for human food systems through the transfer and accumulation of toxic substances within aquatic food chains.

From a regulatory perspective, international and regional frameworks have recognized the hazards posed by several toxic substances associated with industrial and plastic materials. For example, the Stockholm Convention has established binding measures for the elimination or restriction of several highly hazardous persistent substances, including dioxins such as TCDD. However, substances such as DEHP, BPA, and acrylonitrile remain subject to varying degrees of regulation across jurisdictions. The European Union’s REACH regulation has restricted the use of certain phthalates and BPA in specific applications, including food-contact materials and children’s products, whereas other regions continue to permit their use in packaging, electronics, and medical devices. The absence of harmonized global limits complicates enforcement, facilitates the continued movement of contaminated products across borders, and hinders effective risk reduction.

One of the most critical policy gaps lies in substitution management. In several cases, restrictions on a specific plastic-associated chemical have led to the introduction of structurally similar replacements, such as BPAF and other bisphenol analogues or alternative phthalates, whose toxicological profiles remain insufficiently characterized but may involve comparable mechanisms of action. Without mandatory premarket toxicological screening and lifecycle assessments, such substitutions risk perpetuating similar hazards under different chemical names. This issue is particularly relevant to the plastics and materials industry, where functional requirements often rely on plasticizers, stabilizers, and flame retardants with poorly characterized long-term effects. Given the persistence, bioaccumulation potential, and transboundary movement of these substances, source control remains one of the most effective mitigation strategies. Policies should prioritize the reduction and elimination of hazardous plastic-associated chemicals from production streams, the development and adoption of safer material alternatives, and the application of green chemistry principles in polymer synthesis. Complementary measures should include stricter monitoring of industrial effluents, mandatory reporting of hazardous chemical releases, and expansion of analytical surveillance programs for environmental matrices and human biomonitoring.

## 4. Conclusions

The reviewed occurrence data establish that the four compounds follow distinct environmental trajectories reflecting their physicochemical properties: TCDD persists in soil and sediment at contaminated sites for decades, DEHP accumulates in soil near industrial areas while human biomonitoring shows measurable decline after regulatory action, BPA concentrates in densely industrialized regions even as environmental levels fall, and ACN remains a localized contaminant owing to its volatility and rapid biodegradation at low concentrations. These patterns confirm that regulation measurably reduces exposure, but persistence and continued production sustain widespread low-level contamination.

Toxicological findings converge on oxidative stress and mitochondrial dysfunction as the core mechanisms of harm, which are reached through compound-specific signaling nodes: AhR for TCDD, PPAR and the Nrf2/HO-1 axis for DEHP, estrogen receptor-dependent and -independent pathways for BPA, and TLR4/NF-κB signaling for ACN. Through these mechanisms, adverse effects arise in cytotoxic, neurotoxic, cardiotoxic, reproductive, and immune endpoints at environmentally relevant concentrations, and some effects, notably the reproductive ones, extend across generations. Chronic low-dose exposure therefore constitutes a genuine risk profile that high-dose hazard thresholds do not capture. Expression of HSP47 and HSP70 provides an early, dose-responsive biomarker of cellular stress that precedes overt tissue damage, and human biomonitoring programs demonstrate that exposure trends can be tracked and verified over time.

The reviewed findings also highlight the importance of integrating toxicological endpoints into material policies. While performance and durability remain key selection criteria in material design, regulatory frameworks should also incorporate data on endocrine disruption, reproductive toxicity, and chronic immunotoxicity. This integration can be supported by the development of predictive in vitro assays and computational toxicology tools that allow for early hazard identification before materials reach large-scale production.

## Data Availability

No new data were created or analyzed in this study. Data sharing is not applicable to this article.

## Abbreviations

ACN	Acrylonitrile
ABS	Acrylonitrile butadiene styrene
AChE	Acetylcholinesterase
AKT	Protein kinase B
BDNF	Brain-derived neurotrophic factor
BCL-2	B-cell lymphoma 2
BCL-XL	B-cell lymphoma-extra large
BID	BH3-interacting domain death agonist
BPA	Bisphenol A
BPAF	Bisphenol AF
BOD	Biochemical oxygen demand
CAT	Catalase
CHMS	Canadian Health Measures Survey
CMV	Cytomegalovirus
COD	Chemical oxygen demand
DEHP	Di(2-ethylhexyl) phthalate
DLEC	European sea bass embryonic cells
DO	Dissolved oxygen
EPA	Environmental Protection Agency
ER	Estrogen receptor
ERK	Extracellular signal-regulated kinase
GBS	Group B Streptococcus
GSH	Glutathione
HEK-293	Human embryonic kidney 293 cells
HEPG2	Human hepatocellular carcinoma cells
HELA	Human cervical cancer cells
HSFs	Heat shock factors
HSPs	Heat shock proteins
HSR	Heat shock response
IC_50_	Half-maximal inhibitory concentration
ICAM-1	Intercellular adhesion molecule 1
IHD	Ischemic heart disease
IUGR	Intrauterine growth restriction
LH	Luteinizing hormone
LOAEL	Lowest-observed-adverse-effect level
MDA	Malondialdehyde
MECPP	Mono(2-ethyl-5-carboxypentyl) phthalate
MEHHP	Mono(2-ethyl-5-hydroxyhexyl) phthalate
MEOHP	Mono(2-ethyl-5-oxohexyl) phthalate
MEHP	Mono(2-ethylhexyl) phthalate
MTT	3-(4,5-Dimethylthiazol-2-yl)-2,5-diphenyltetrazolium bromide
NHANES	National Health and Nutrition Examination Survey
NTP	National Toxicology Program
PARP	Poly(ADP-ribose) polymerase
PCDD	Polychlorinated dibenzo-p-dioxin
PPAR	Peroxisome proliferator-activated receptor
POPs	Persistent organic pollutants
PSD-95	Postsynaptic density protein 95
PVC	Polyvinyl chloride
SH-SY5Y	Human neuroblastoma cell line
SOD	Superoxide dismutase
TBARS	Thiobarbituric acid reactive substances
TCDD	2,3,7,8-Tetrachlorodibenzo-p-dioxin
WHO	World Health Organization
